# Antibiotic Susceptibility Patterns for Carbapenem-Resistant Enterobacteriaceae

**DOI:** 10.1155/2023/8920977

**Published:** 2023-02-20

**Authors:** Shahnaz Armin, Fatemeh Fallah, Abdollah Karimi, Fereshteh Karbasiyan, Masoud Alebouyeh, Sedigheh Rafiei Tabatabaei, Maryam Rajabnejad, Roxana Mansour Ghanaie, Seyed Alireza Fahimzad, Nafiseh Abdollahi, Hannan Khodaei, Leila Azimi

**Affiliations:** ^1^Pediatric Infections Research Center, Research Institute for Children's Health, Shahid Beheshti University of Medical Sciences, Tehran, Iran; ^2^Department of Pediatric Gastroenterology and Hepatology, Iran University of Medical Sciences, Tehran, Iran

## Abstract

Carbapenem is a broad-spectrum beta-lactam antibiotic considered the last choice for the treatment of antibiotic-resistant Gram-negative bacteria. Thus, the increasing rate of carbapenem resistance (CR) in Enterobacteriaceae is an urgent public health threat. This study aimed to evaluate the antibiotic susceptibility pattern of carbapenem-resistant *Enterobacteriaceae* (CRE) to new and old antibiotics. In this study, *Klebsiella pneumoniae*, *E. coli*, and *Enterobacter* spp. were collected from 10 hospitals in Iran for one year. CRE is recognized by resistance to meropenem and/or imipenem disk after identification of the collected bacteria. Antibiotic susceptibility of CRE against fosfomycin, rifampin, metronidazole, tigecycline, and aztreonam was detected by disk diffusion method and colistin by MIC. In this study, 1222 *E. coli*, 696 *K. pneumoniae*, and 621 *Enterobacter* spp. were collected from 10 hospitals in Iran in one year. Fifty-four *E. coli* (4.4%), 84 *K. pneumoniae* (12%), and 51 *Enterobacter* spp. (8.2%) were CRE. All CRE strains were resistant to metronidazole and rifampicin. Tigecycline has the highest sensitivity on CRE and levofloxacin for *Enterobacter* spp. Tigecycline showed an acceptable effectiveness rate of sensitivity on the CRE strain. Therefore, we suggest that clinicians consider this valuable antibiotic to treat CRE.

## 1. Introduction

Carbapenems were considered as the last choice antibiotic for the treatment of bacteria, subsequent to the global increase in the rate of multidrug-resistant (MDR) Gram-negative bacteria, in recent years. The development of carbapenem-resistant (CR) Enterobacteriaceae is a greatly challenging problem because there is no noticeable next line of antibiotics for the treatment of these kinds of antibiotic-resistant organisms [1, 2]. Thus, the CDC announces carbapenem-resistant Enterobacteriaceae (CRE) as an urgent public health threat. Based on the data, antimicrobial-resistant organisms, such as CRE, can spread among healthcare providers, patients, and facilities when they are infected or colonized patients [3]. Therefore, following an antimicrobial resistance (AMR) change in each region is essential for determining prevention and therapeutic strategies.

Growing resistance to carbapenems has increased the need for the development of new antibiotics. On the other hand, nowadays, production of new antibiotic has declined and The Infectious Disease Society of America (IDSA) announced that the “pharmaceutical pipeline is drying up” [4]. In this situation, the best way to combat resistant organisms and save the patients' life is accurate usage of available antibiotics.

To date, laboratories do not have any special protocol for the detection of the antibiotic susceptibility of these organisms. Also, the best clinical management of CRE infections has not been established yet because clinical trials have never shown the optimal treatment strategies. Moreover, based on evidence, CRE can be susceptible to tigecycline and aminoglycosides (mostly gentamicin) and some old drugs such as fosfomycin and colistin. Therefore, clinicians can use these antibiotics as their drugs of choice for the treatment of infections caused by such bacteria.

We conducted this study to evaluate antibiotic susceptibility pattern of CRE to new and old antibiotics so that our knowledge about treatment options for CRE infection is increased.

## 2. Material

### 2.1. Setting and Sampling

In this study, we worked on *K. pneumoniae*, *E. coli*, and *Entobacter* spp. as the most important *Entrobacteriaceae* family isolated from 10 hospitals in 9 cities in different areas in Iran during one year. These cities included Tehran, Sanandaj, Esfahan, Gorgan, Tabriz, Hamedan, Zahedan, Mashhad, and Ahvaz. The specimens were sent to the microbial laboratory from different wards of the hospitals such as emergency, ICU, ENT, gastrointestinal ward, transplant, and urology wards. The sources of specimens such as the wound, blood, urine, and CSF were various as well.

### 2.2. Bacterial Identification

Bacterial strains were identified by conventional biochemical and microbiological methods including Gram stain, TSI, citrate, SIM, and MR/VP. Antibiotic susceptibility was determined after confirmed identification.

### 2.3. Antibiotic Susceptibility Testing

The CRE strains were recognized by the determination of resistance to meropenem (10 *μ*g) and imipenem disk (10 *μ*g), based on CLSI 2020 [5]. We evaluated antibiotic susceptibility of CRE to fosfomycin, rifampin, metronidazole, tigecycline, and azteronam by disk diffusion method and MIC for colistin [4] which were evaluated by microdilution method, based on CLSI protocol 2020 [4]. Susceptibility to tigecyline was evaluated according to the break point of the Food and Drug Administration (FDA) [6] because it was not included in the CLSI protocol.

## 3. Statistical Analysis

Categorical variables were reported as frequency (percentage). All analyses were performed using the Statistical Package for Social Sciences (SPSS Inc., version 21.0, Chicago, IL, USA).

## 4. Result

The specimens were collected (46% female and 53% male). Also, the age range was between one and 92 years old. 1222 *E. coli*, 696 *K. pneumoniae*, and 621 *Enetrobacter* spp. were collected from 10 included hospitals in Iran in one year.

Fifty-four (4.4%), 84 (12%), and 51 (8.2%) *E. coli*, *K. pneumoniae,* and *Enterobacter* spp. were CRE, respectively; then, they were enrolled in this study. All CRE strains were resistant to metronidazole and rifampicin. The colistin-resistant rate was 67.2% in the included bacteria. The tigecycline was the most effective antibiotic in the current study with 62% sensitivity (Table 1). 32.8% of the strains showed a moderate effect on CRE strains; this was also the most prevalent percentage of the moderate effectiveness of the considered antibiotics in this study.

## 5. Discussion

Antimicrobial resistance is one of the threats to the public health systems in the world. Based on the literature, the burden of Gram-negative resistance bacteria as a major cause of hospital-acquired infection is increasing day by day [7–9]. For years, carbapenem was used as one of the last lines of antibiotic therapy for resistant bacteria, especially ESBL producing [10, 11]. Resistance to carbapenem leads to serious challenges for physicians and push them to use colistin as the last option, despite of its nephrotoxic effect. On the other hand, increased use of colistin can lead to a repetition of the carbapenems resistance tragedy. In this condition, physicians should use combination therapy, although there is no approved protocol for this situation and use of this type of treatment can increase the rate of side effects.

Subsequently, clinicians have been forced to reexamine old and new agents for CRE which have been rarely used because of their side effect or effectiveness, such as polymyxins, fosfomycin, rifampin, tigecycline, levofloxacine, azteronam, and metronidazol. In this study, all CREs showed resistance to metronidazole and rifampicin. In another study, researchers pointed to metronidazol as a part of combination therapy, but our data indicated that they were not effective in vitro [12]. Zhang et al. reported carbapenem plus metronidazole combined can be favorable therapeutics in *K. pneumoniae*-induced liver abscess. [13].

Various studies have used rifampin as combination therapy for drug-resistant Entrobacteriaceae [12]. Tangden et al. suggested the combination of rifampin-meropenem- colistin as the best regimen. Data showed a synergistic and bactericidal effect of this combination against *K. pneumoniae*-producing carbapenemase enzymes [14].

Aztreonam was approved in 1986. It is a member of the monobactam antibiotics family, which is stable to OXA-48 enzymes and metallo-beta-lactamase (MBL) activity but is hydrolyzed by serine *β*-lactamases [15]. Thus, it has been used as a treatment for some kinds of carbapenem-resistant Entrobacteriaceae. In our study, we detected 90.5% resistance pattern against aztreonam. Sader et al. identified aztreonam resistant rate is 10.9% in different carbapenemase-producing bacteria [16]. This difference in aztreonam resistance rate may be because of different carbapenemase-producing enzymes bacteria in the two studies.

That our strain producing serine *β*-lactamases, such as extended-spectrum *β*-lactamases, AmpC, and KPC in contrast of carbapenemase-producing bacteria were surveyed in Helio et al. study.

Some reports show that oral and intravenous fosfomycin is an acceptable option for treatment of CRE [12]. Snayd et al. reported that 28.3% of carbapenem-resistant Entrobacteriaceae were fosfomycin nonsusceptible by CLSI criteria; in our study, this rate was 41.2% [17].

This disagreement may be due to the source of specimens, the pattern of antibiotic usage in different geographical areas, and the detection of resistance organisms by the fosfomycin disk method which is complicated because of the scattered colony observed inside the inhibition zone that may decrease the accuracy of this method [18]. In addition, some researchers reported that disk diffusion and Etest have poor agreement with fosfomycin agar dilution as the reference method [19]. In our study, the resistance rate to levofloxacin was 74%; other studies indicate that CRE often exhibits resistance to structurally unrelated antimicrobial classes such as fluoroquinolones [12]. Thus, we predicted this high rate of resistance.

Colistin is the last line of antibiotics for the treatment of CRE [20]. In this study, we detected a high rate of colistin resistance. To the best of our knowledge, there is no other study on clinical isolates using the standard method and no multicenter study on the frequency of colistin resistance CRE in Iran. Some studies reported the low rate of colistin-resistant Entrobacteriaceae, such as Aghapour et al.'s study which reported 3.33% resistance rate from 900 clinical isolates [21]. While the rate was so low, the detection method was not based on CLSI recommendation. Pishnian et al. revealed 0.96% colistin-resistant Entrobacteriaceae from poultry, not clinical human specimens [22].

On the global surveillance program which was done in 2016, the researcher studied the collected *Entrobacteriaceae* from 39 countries and identified colistin-resistant bacteria. Unfortunately, there was no data from Iran [23]. Haeili et al. worked on clinical specimens to find colistin-resistant *Entrobacteriaceae* in one of the largest referral hospitals in Iran for 2 years (2016–2017). They reported just 20 *k. pneumoniae* colistin-resistant isolates. The data show the rate of colistin resistance was not so high. Unfortunately, in our study, we face with some big challenges with these threatening organisms by 67.2% resistance rate of colistin. Limitation for choosing the best antibiotics is due to the increasing rate of colistin resistance and spreading them around different parts of Iran [24].

Tigecycline has a wide spectrum of activity on various Gram-positive, Gram-negative, and anaerobic organisms as the primary drug in the glycylcycline class [25]. Although the FDA [6] endorses cut-off point for interpreting the result of tigecycline on Entrobacteriaceae family, CLSI does not recommend any protocol [5]. In our study, based on FDA recommendation, we found only 5.2% tigecycline-resistant CRE. To the best of our knowledge, this is the first report to evaluate tigecycline susceptibility on CRE in Iran. Stefani and Dowzicky reported the activity of tigecycline against Gram-negative organisms in Italy between 2012 and 2014. They showed increased resistance to tigecycin in this period from 7.4 to 15.8 in MDR *K. pneumoniae* [26].

Morrill et al. suggest tigecycline as an option for treatment of CRE [12]. Resistance to tigecycline has happened during therapy of the CRE [27]. Therefore, researchers recommend that tigecycline should be used in combination therapy to treat CRE infections to overcome this pitfall.

Because of its bacteriostatic activity and low steady-state concentrations at usual dosing of tigecycline, it is generally not suggested for the treatment of bacteremia as a single therapy [28, 29].

In areas with limited availability of effective drugs for CRE infections and high rate of susceptibility to tigecycline, like our situation, it should not be restricted carelessly.

## 6. Conclusion

As using colistin is the last line of treatment of the CRE infection, the high rate of colistin resistance in this study is alarming for the healthcare system in Iran. Fortunately, tigecyclin shows an acceptable rate of sensitivity on the CRE strain, which has not been considered before. Thus, we suggest that clinicians should use this valuable neglected antibiotic in combination protocol, and also, it should be better that clinicians do not use broad-spectrum antibiotics easily, especially in experimental treatment.

## 7. Limitations

The limitation of this study was that we did not clarify whether the isolated bacteria were the cause of colonization or infection.

## Figures and Tables

**Table 1:** Antibiotic susceptibility patterns of CRE.

	Colistin			Aztreonam			Levofloxacin			Fosfomycin			Tigecycline		
	*S*	*I*	*R*	*S*	*I*	*R*	*S*	*I*	*R*	*S*	*I*	*R*	*S*	*I*	*R*
*E. coli* (no. 54)	22 (40.8)	—	32 (59.2)	8 (14.8)	1 (1.9%)	45 (83.3)	9 (16.7)	—	45 (83.3)	32 (59.2)	4 (7.5)	18 (33.3)	38 (70.4)	14 (25.9)	2 (3.7)
*K. pneumoniae* (no. 84)	33 (39.2)	—	51 (60.8)	2 (2.3)	1 (1.3)	81 (96.4)	11 (13.1)	—	73 (86.9)	26 (30.9)	18 (21.4)	40 (47.6)	50 (59.6)	31 (36.9)	3 (3.5)
*Enterobacter* spp. (no. 51)	7 (13.8)	—	44 (86.2)	5 (9.8)	1 (2)	45 (88.2)	29 (56.9)	—	22 (43.1)	27 (52.9)	4 (7.6)	20 (39.2)	29 (56.9)	17 (33.3)	5 (9.8)
Total (no. 189)	62 (32.8)	—	127 (67.2)	15 (8)	3 (1.5)	171 (90.5)	49 (26)	—	140 (74)	85 (45)	26 (13.8)	78 (41.2)	117 (62)	62 (32.8)	10 (5.2)

## Data Availability

The data were obtained from the study. Requests for the data will be considered after the publication of this article.
